# The benefits of trastuzumab in the treatment of HER2+ breast cancer as a function of exposure time

**DOI:** 10.3332/ecancer.2022.1347

**Published:** 2022-01-20

**Authors:** Felipe Andrés Cordero da Luz, Eduarda da Costa Marinho, Camila Piqui Nascimento, Lara de Andrade Marques, Patrícia Ferreira Ribeiro Delfino, Rafael Mathias Antonioli, Marcelo José Barbosa Silva, Rogério Agenor de Araújo

**Affiliations:** 1 Center for Cancer Prevention and Research, Uberlandia Cancer Hospital, Av Amazonas nº 1996, Umuarama, Uberlândia, Minas Gerais, 38.405-302, Brazil; 2 Laboratory of Tumor Biomarkers and Osteoimmunology, Institute of Biomedical Sciences, Federal University of Uberlandia, Av Pará nº 1720, Bloco 6T, room 07, Umuarama, Uberlândia, Minas Gerais, 38.405-320, Brazil; 3 Faculty of Medicine, Federal University of Uberlandia, Av Pará nº 1720, Bloco 6T2u, room 07, Umuarama, Uberlândia, Minas Gerais, 38.400-902, Brazil; a https://orcid.org/0000-0002-9381-4913; b https://orcid.org/0000-0002-1307-9104; c https://orcid.org/0000-0002-0955-8559; d https://orcid.org/0000-0002-2734-8352; e https://orcid.org/0000-0002-2196-9318; f https://orcid.org/0000-0003-3886-1562; g https://orcid.org/0000-0002-5807-4286; h https://orcid.org/0000-0003-4653-6786

**Keywords:** antineoplastic agents, breast neoplasms, ErbB-2 receptor, prognosis, trastuzumab

## Abstract

**Background:**

Breast cancer is a heterogeneous disease with overexpression of several receptors, such as human epidermal receptor 2 (HER2), which is a prognostic and predictive biomarker for treatment with the anti-HER2 monoclonal antibody trastuzumab. This study aimed to test the contribution of this regimen in patients with overexpression/amplification of HER2 for periods shorter than the 1-year treatment recommendation.

**Methods:**

A retrospective single-centre study involving 155 patients with non-metastatic (stages I–III) invasive ductal HER2+ breast carcinoma, with a median follow-up of 48.9 months after completion of adjuvant therapy, except endocrine therapy.

**Results:**

About 60% of patients received trastuzumab therapy for a median time of 365 days. Although the use of trastuzumab for a short period has provided some benefit, analyses of survival with a continuous dependent variable have revealed a minimum time for improved survival. In the multivariate analysis by Cox regression, trastuzumab use duration exceeding 9 weeks resulted in protection against distant metastasis (adjusted HR: 0.307 (0.139–0.678), *p* = 0.004), disease progression (adjusted hazard ratio (HR) 0.353 (0.175–0.714), *p* = 0.004) and death (adjusted HR: 0.267 (0.105–0.678), *p* = 0.005), being superior to multimodal systemic therapy with chemotherapy and to endocrine therapy without trastuzumab, but inferior to almost 1 year of administration of this monoclonal antibody, especially regarding overall survival (adjusted HR: 0.203 (0.069–0.596), *p* = 0.004).

**Conclusion:**

Despite showing some benefits, the protective effect derived from a suboptimal time of trastuzumab exposure is inferior to the standard course of 1 year.

## Background

Breast cancer is a highly heterogeneous disease, classified by the TNM (T – tumour; N – lymph node metastasis; M – distant metastasis) system, histological grade and expression of hormone receptors (oestrogen – ER and progesterone – PR) and human epidermal receptor 2 (HER2) [1–4]. The overexpression (intense membrane staining of >10% of tumour cells by immunohistochemistry) and/or amplification of the ERBB2 gene locus, which codes for HER2, is necessary for classification as HER2+ tumours [5].

Irrespective of the expression of hormone receptors, 15%–20% of breast cancers are of the HER2+ subtype [3, 6]. Historically, HER2 overexpression/amplification is related to the worst outcome in breast cancer patients [7]. However, the development of targeted trastuzumab therapy has caused a revolution in the prognosis of these patients [8]. Trastuzumab is a neutralising monoclonal antibody that mainly leads to HER2 blockade, by impeding its signalling pathway activation, which is associated with cell proliferation and survival [9]. Thus, its amplification/overexpression leads to rapid tumour progression [9]. This is corroborated by the fact that HER2 prevails as a worse prognostic factor in patients who did not receive adequate systemic therapy [10].

The availability of this therapy caused such a revolution that it led to the overexpression/amplification of HER2 as a downstaging criterion in the recent staging manual (eighth edition) by the American Joint Committee on Cancer (AJCC) [11, 12]. However, its high cost over an entire 1-year course of treatment [9] hinders both its implementation and its continuous supply in public health services of developing countries, such as Brazil [13, 14]. Thus, the search for a shorter time of exposure to trastuzumab, with equivalent benefits, is essential to be able to offer this indispensable therapy uninterruptedly and universally to all these women, so that they do not suffer the deleterious effect that the amplification/overexpression of HER2 has on their prognosis [9, 14]. In this regard, despite some contradictions, some clinical trials have demonstrated that the benefit of a shorter regimen with trastuzumab did not lose effectiveness [9], generating potential savings and availability to a greater number of patients [14].

This retrospective study aimed to analyse the benefit of trastuzumab in the treatment of patients with breast cancer undergoing adjuvant treatment attended in a public health unit in Brazil, and test whether there is a minimum exposure time, less than 1 year, which generates protection.

## Methods

### Study design

A retrospective observational study of patients with breast cancer treated at the oncology sector of the Federal University of Uberlandia between January 1999 and November 2021 was performed. Based on the result of the anatomopathological examination, and not on the medical notes, all patients were reclassified in their pathological TNM according to the Seventh Edition of the AJCC [15]. For prognostic analysis, both T and N classifications were obtained by clinical and pathological examinations, independently if the patient received neoadjuvant therapy. Patients’ ages were classified into two categories according to the following criteria: <70 years and ≥70 years for overall survival (OS) due to shorter life expectancy, higher risk of all-cause death and having received fewer treatments [16, 17].

The systemic treatment was considered adequate whenever patients received treatment as indicated by current guidelines. A systemic treatment was considered correct when patients received complete course of chemotherapy, according to chemotherapy schema, and trastuzumab (except when T < 1 cm – T1a/T1b), for a minimum time of 1 year, and received endocrine therapy when tumours expressed at least one positive hormone receptor [1, 2]; hormone receptor expression was considered positive when at least 1% of tumour cells were detected as expressing by the immunohistochemistry approach [4]. Trastuzumab exposure/administration time was measured in days between the date of the first dose and the date of the last dose but the exposure to at least 14 cycles of trastuzumab was also considered adequate.

### Ethical factor

This study was approved by the Human Research Ethics Committee of the local Institution (protocol number 803.826/14) and followed all the ethical principles of the Declaration of Helsinki and its subsequent amendments or comparable ethical standards. The informed consent form was waived, according to the type of study performed.

### Inclusion and exclusion criteria

This study enrolled female patients with invasive ductal carcinoma of no special type/no otherwise specified histology with overexpression/amplification of HER2. Patients were excluded according to the following criteria: synchronic metastatic disease (initial diagnosis or within 6 months); missing histopathological data (surgical margin or histological grade); incomplete immunohistochemistry (absence of ER or PR) and/or indeterminate HER2 (2+ with indeterminate/without hybridisation method); failure to perform surgery; neoadjuvant radiation therapy; the presence of a special component (papillary, mucinous, cribriform, etc.); lysed/destroyed tumour; bilateral cancer; more than one primary cancer; tumour progression during adjuvant treatment, except endocrine therapy; unreported cause of death and follow-up time less than 180 days from diagnosis to event.

From a total of 2,580 medical records, 1,685 were excluded for being metastatic or *in situ* tumours, or lack of adequate pathological examination, lack of surgery, neoadjuvant radiation therapy, development of another cancer and/or bilateral/contralateral breast cancer. Of the remaining 895 medical records, 737 were excluded for being Luminal A or B or TN. Three patients were subsequently excluded due to tumour progression during chemotherapy, trastuzumab or radiation therapy course.

### Outcomes

Distant metastasis-free survival (DMFS) was defined as the time from diagnosis to development of contralateral lymph node metastasis and/or to any distant organ. Disease-free survival (DFS) was considered the time from diagnosis to development of any relapse (local, regional or distant metastasis) or death from any cause. OS was defined as the time from diagnosis to death from any cause.

### Statistical analysis

The Kolmogorov–Smirnov normality test, descriptive analyses, cross-table analysis of concordance (Cohen’s Kappa), Kaplan–Meier and Cox regression were performed on the software IBM SPSS v25.0. The survival curves with continuous predictor and their optimal cutoff point were established using the software Jamovi v1.6.5.0. In all analyses, statistical significance was defined as *p* < 0.05.

The Kaplan–Meier estimator was used to analyse the proportionality of risks as a prerequisite for considering the variable in the Cox regression model in multivariate analysis. The time-dependent Cox regression model was used to test the influence of time from diagnosis to the end of adjuvant therapy, except endocrine therapy, on outcomes. Multivariate Cox regression was performed using the Stepwise Forward Wald method with an entry *p*-value of 0.25 [18] and output *p*-value of 0.10 for analysis of independent prognosis factor with superiority.

## Results

Of the included patients (*n* = 155), only 96 (61.9%) received trastuzumab, for a median time of 365 days, but only 70 (45.2 %) received an adequate treatment according to the 2007 St. Gallen Consensus standard guidelines [19]. All characteristics are depicted in Table 1.

### Trastuzumab treatment has a positive impact on a patient’s prognosis

The added benefit of trastuzumab treatment was later tested in relation to the endpoints of distant metastasis, any progression and death.

First, it was tested whether the time from diagnosis to end of therapy could imply immortal time bias. By time-dependent Cox regression, this time had a non-significant impact only on disease progression (hazard ratio (HR): 150.357 (0.738–30,616.233), *p* = 0.065). Due to this result and theoretical background (unpublished results), only the time from the end of adjuvant (surgery, chemotherapy, trastuzumab or radiation therapy) to the observed outcome was further considered.

**Figure 1.**
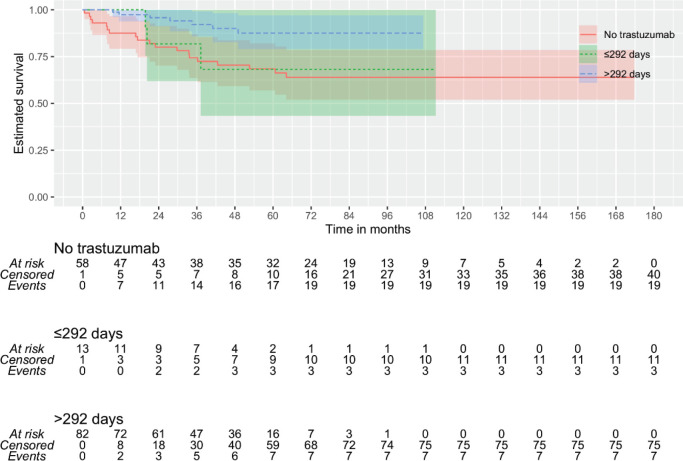
KMunicate plot for DMFS according to the time of administration of trastuzumab. Patients of the group of ≤292 days of exposition included 59 of no treatment and 15 with less or equal to 292 days of the exposition.

**Figure 2.**
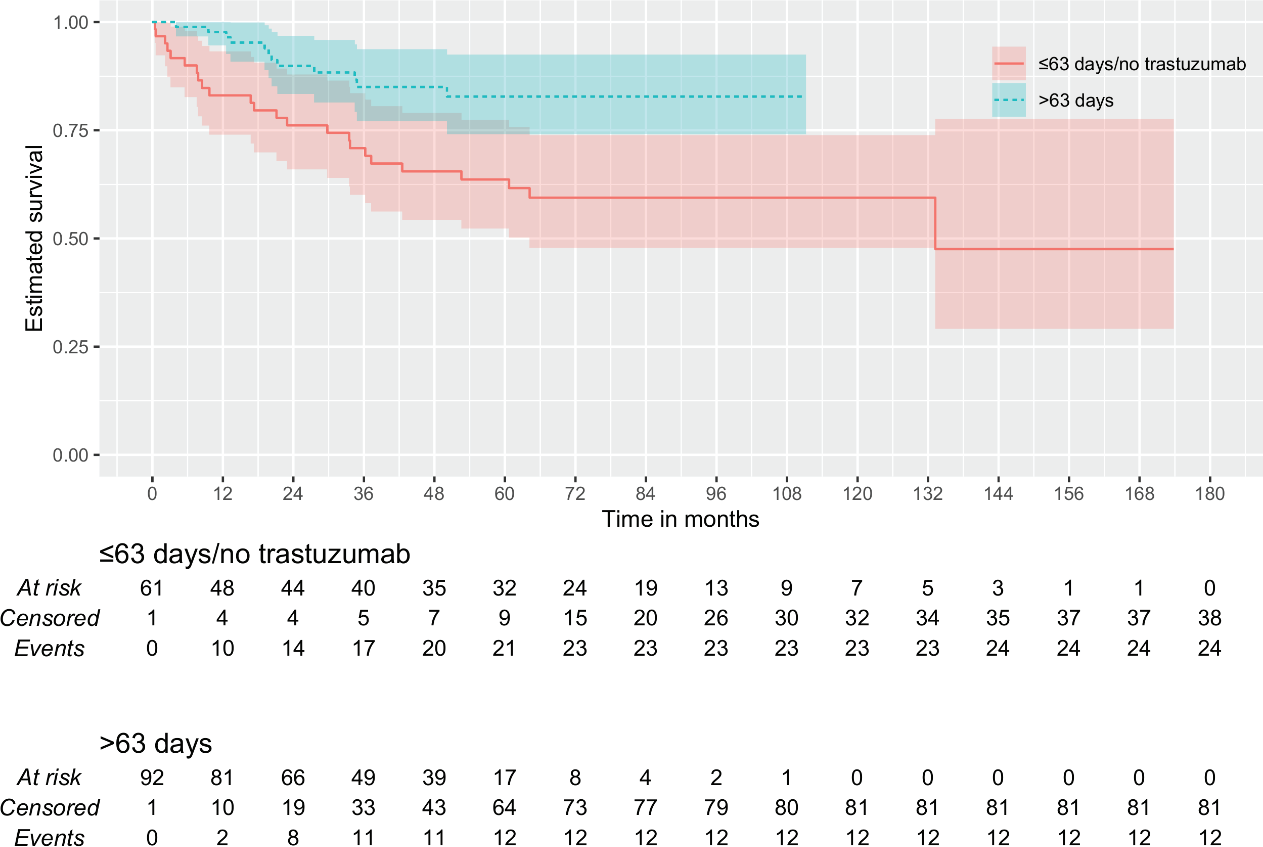
KMunicate plot for DFS according to the time of administration of trastuzumab. Patients of the group of ≤63 days of exposition included 59 of no treatment and 3 with less or equal to 63 days of the exposition.

**Figure 3.**
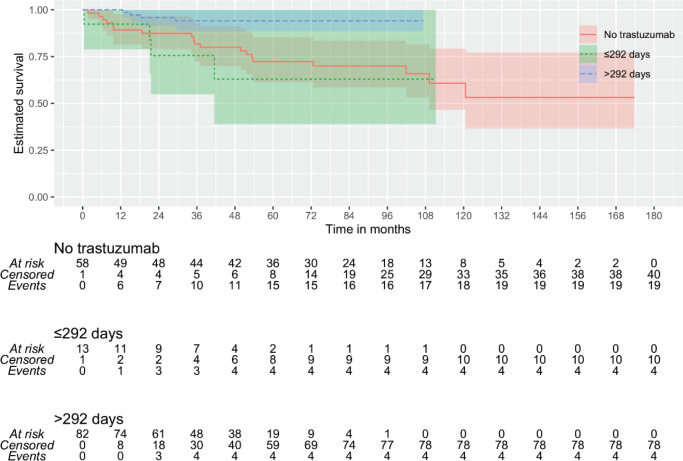
KMunicate plot for OS according to the time of administration of trastuzumab. Patients of the group of ≤292 days of exposition included 59 of no treatment and 15 with less or equal to 292 days of the exposition.

Then, it was tested whether the duration of trastuzumab exposure could impact outcomes. By continuous survival analysis, it was observed that the duration of trastuzumab exposure has a significant impact on DMFS (HR: 1.00 (0.99–1.00), *p* = 0.011) and OS (HR: 1.00 (0.99–1.00), *p* = 0.008) and is inversely associated with the outcome when there were more than 292 days of trastuzumab exposure. On the other hand, although also significant for DFS (HR: 1.00 (1.00–1.00), *p* = 0.018), it was inversely associated with the outcome when there were more than 63 days of trastuzumab exposure. Kaplan–Meier plots are depicted in Figures 1–3. The median time of survival (50%) was achieved only for patients with up to 63 days/no exposure to Trastuzumab regarding DFS (133.23 months).

Because of these discrepancies, patients were categorised according to time of Trastuzumab administration: 1) ≤63 versus >63 days (9 weeks) of exposure and 2) ≤292 versus >292 days of exposure. Additionally, it was tested whether any used time of exposure – either more than 84 days (12 weeks) or at least 6 months of exposure – could provide some benefit. An exposure of fewer than 292 days did not differ significantly from no exposure at all (Figures 1 and 3). The patients with no trastuzumab exposure were clustered together with patients receiving suboptimal time of trastuzumab exposure.

Based on these cutoffs, 93 patients (60.0%) were classified as receiving trastuzumab for more than 63 days, 91 (58.7%) patients as receiving it for 12 weeks or more, 86 (55.5%) as receiving this treatment for 6 months or more and 82 (52.9%) as receiving it for more than 292 days. Their counterparts included 62 (59 no treatment and 3 with less than or equal to 63 days of exposure), 64 (59 no treatment and 5 a maximum of 12 weeks of exposure), 69 (59 no treatment and 11 with less than 6 months of exposure) and 73 (59 no treatment and 15 with less than or equal to 292 days of exposure) patients, respectively.

By stepwise analysis, the trastuzumab exposure exceeding 292 days resulted in increased capacity for protecting against distant metastasis (Table 2), and death (Table 3), and any progression. Furthermore, it was observed that the trastuzumab exposure for a longer time (>292 days) has a stronger impact than the multimodal treatments by the St. Gallen Consensus (Tables 2–4), though a moderate concordance was observed between trastuzumab administration and adequate treatment (Cohen’s Kappa: 0.795, *p* < 0.0005).

The trastuzumab exposure time obtained as optimal was very close to that recommended as standard (1 year), except for DFS. In order to test whether a shorter period offers similar protection, analyses were redone, but without inserting the optimal cutoff of each outcome in the model. This time, exposure for more than 63 days resulted in greater protection in stepwise multivariate Cox regression for DMFS (adjusted HR: 0.307 (0.139–0.678), *p* = 0.004) and for OS (adjusted HR: 0.267 (0.105–0.678), *p* = 0.005), while an exposure exceeding 292 days was associated with improved DFS (adjusted HR: 0.358 (0.172–0.748), *p* = 0.006).

The former consensus preconised trastuzumab except in patients with T1a/T1b, N0 disease [19]. Therefore, we excluded these patients from analysis (*n* = 8). Continuous survival analysis showed different cutoffs, of >63 days for DMFS (*p* = 0.0005) and DFS (*p* = 0.007), and of >292 days for OS (*p* = 0.0004).

DMFS, as the only cutoff point that differed, was tested for its ability to impact the multivariate analysis. By stepwise Cox regression, the cutoff based on 9 weeks of exposure was retained in the model, with improved DMFS (adjusted HR: 0.287 (0.130–0.638), *p* = 0.002), and was not substantially different from the benefit associated with >292 days of exposure (adjusted HR: 0.269 (0.114–0.634), *p* = 0.003).

As the analyses included patients with no exposure to trastuzumab, analyses were performed again excluding these patients. No impact was observed regarding DMFS (HR: 1.00 (0.99–1.00), *p* = 0.565) nor DFS (HR: 1.00 (0.99–1.00), *p* = 0.422), but OS was found to be inversely associated with an exposure greater than 292 days (HR: 0.99 (0.99–1.00), *p* = 0.042). The exposure time of >292 days resulted in improved OS (adjusted HR: 0.162 (0.040–0.652), *p* = 0.010) by multivariate Cox analysis.

## Discussion

Trastuzumab is a turning point in treating breast cancer patients with HER2 overexpression/amplification [8]. Its implementation leads to improved DMFS [20], DFS [8, 20], OS [8] and even locoregional relapse-free survival [20]. This retrospective study demonstrated that trastuzumab is the leading systemic therapy associated with improved outcomes, even compared to an adequate multimodal systemic treatment.

In Brazil, the implementation of trastuzumab in the treatment guidelines of the public system only took place in 2013 [21]. Even before this occurred, discussion of the economic impact of this therapy in the recommended complete 1-year course suggested the implementation of shorter times [14]. In fact, the implementation of trastuzumab can increase the cost of treating a patient by approximately 300% [13]. Additionally, even with a progressive decrease in the cost of trastuzumab, the augmented number of cases makes this therapy increase the economic burden of treating neoplasias [22]. Thus, searching for shorter optimal times of administration is critical. We observed that an exposure time greater than 63 days is associated with improved outcomes, especially those associated with relapses (DMFS and DFS).

Several clinical trials reported non-inferiority of a shorter time of trastuzumab administration compared to 12 months, such as 6 months [23], 12 weeks [24] and even 9 weeks [25, 26]. Although one clinical trial failed to observe non-inferiority of 6 months compared to 1 year of trastuzumab administration [27], a recent meta-analysis reported non-inferior protection of 6 months compared to 1 year [28]. Interestingly, the optimal cutoff observed in this study was of 9 weeks (63 days). This cutoff provided protection against disease progression superior to 12 weeks and 6 months, suggesting that patients with an exposure between 9 weeks and 12 weeks or 6 months still derived benefit from trastuzumab exposure. This effect was more evident after excluding T1a/T1b patients, which did not indicate trastuzumab treatment in the previous guidelines [19]. Indeed, further studies have shown the benefit of adding trastuzumab in patients with T1b (>0.5 to ≤1 cm)-N0 tumours [1], which can be considered a study bias that was also eliminated.

A shorter time of trastuzumab exposure could result in less toxicity but also an increased risk of disease progression [28]. Because the high number of patients who did not receive trastuzumab were grouped together with a small number who received suboptimal treatment, it is difficult to draw any firm conclusions about this. In order to elucidate the potential benefit of these suboptimal treatments, the study design included patients with increasing exposure times, either by cutoff points obtained in our analyses or by data from the literature. In this regard, there was statistical superiority in this type of analysis (Stepwise method) compared to simply any exposure time or no exposure, suggesting that an exposure time of >9 weeks can achieve significant protection against disease progression. However, we observed a benefit loss of 2.3% for DMFS, and of 6.4% for OS, compared to an administration time close to the recommended 1 year. Thus, an entire course of administration (~1 year), compared to less exposure time, could potentially prevent at least 6 deaths per 100 patients treated long-term. In fact, this exposure time was also significant in the analysis considering only patients who received any treatment with trastuzumab in relation to OS.

This study has some limitations, such as the long follow-up time, which implies changes in protocols. And despite the statistical design employed, the impact for a vast majority of patients who received no trastuzumab treatment (95% and 81% in the <63 days and <292 days, respectively) groups makes it difficult to draw any correct conclusions about the effect of shorter exposure times. Despite the representativeness of our setting in a real-world public health service in a developing country, we cannot draw definitive conclusions on efficacy. However, even with significantly different groups, it was possible to observe, among those who received some amount of trastuzumab, that an optimal administration of treatment (~1 year) is a greater protection factor than suboptimal treatments, according to current guidelines, making it inadvisable to shorten its administration.

## Conclusions

The administration of trastuzumab improves survival in non-metastatic breast cancer patients, and is the main therapy for managing HER2-positive overexpressing/amplified tumours. The entire course of trastuzumab administration achieves the maximum benefit, in contrast to the loss of protection for patients receiving suboptimal or no trastuzumab.

## Conflicts of interest

The authors declared that there are no conflicts of interest.

## Funding statement

The authors received no financial support for the research, authorship and/or publication of this article.

## Authors’ contributions

FACL conceived the study. FACL, ECM, CPN, LAM and PFRD designed the methodology and collected the data. FACL performed data analysis. RMA, RAR and MJBS supervised and validated data collection and analysis. FACL wrote the initial manuscript. All authors reviewed and approved the final draft.

## Figures and Tables

**Table 1.** Clinical data of included patients (*n* = 155).

Variable	*N* (%)	Median (minimum– maximum)/mean (± SD)
Time to metastasis	155 (100)	61.87 months (7.00–184.80)
Time to progression	155 (100)	60.07 months (7.00–184.80)
Time to death	155 (100)	65.83 months (7.00–184.80)
		
Time to end of adjuvance	155 (100)	16.93 months (1.40–53.40)
Age	155 (100)	53 years (28–79)
Age		
<70 years	146 (94.2)	
≥70 years	9 (5.8)	
Distant metastasis developed		
No	126 (81.3)	
Yes	29 (18.7)	
Progression		
No	119 (76.8)	
Yes	36 (23.20)	
Deaths		
No	128 (82.6)	
Yes	27 (17.4)	
		
		
		
T (initial)		
T1	37 (23.9)	
T2	63 (40.6)	
T3	30 (19.4)	
T4	25 (16.1)	
N (initial)		
N0	68 (43.9)	
N1	48 (31.0)	
N2	26 (16.8)	
N3	13 (8.4)	
T (pathological)		
T1	55 (35.5)	
T2	77 (49.7)	
T3	13 (8.4)	
T4	10 (6.5)	
N (pathological)		
N0	84 (54.2)	
N1	38 (24.5)	
N2	23 (14.8)	
N3	10 (6.5)	
Histological grade		
G1	6 (3.9)	
G2	106 (68.4)	
G3	43 (27.7)	
Hormone receptor		
Negative	64 (41.3)	
Positive	91 (58.7)	
Trastuzumab (any quantity)		
Did not received	59 (38.1)	
Received	96 (61.9)	365 days (21–502)
Trastuzumab (63 days)		
≤63 days	3 (3.1)	63 days (21–63)
>63 days	93 (96.9)	365 days (71–502)
Trastuzumab (12 weeks)		
≤12 weeks	5 (5.2)	63 days (21–82)
>12 weeks	91 (94.89)	365 days (126–502)
Trastuzumab (6 months)		
<6 months	10 (10.4)	104 days (21–169)
≥6 months	86 (89.6)	367 days (216–502)
Trastuzumab (292 days)		
≤292 days	59 (38.1)	142.5 days (21–292)
>292 days	96 (61.9)	369 days (296–502)
Endocrine therapy		
Negative	8/91 (8.8)	
Positive	83/91 (91.2)	
Chemotherapy		
No	10 (6.5)	
Neoadjuvant	50 (32.3)	
Adjuvant	95 (61.2)	
Systemic treatment (St. Gallen)		
Inadequate	85 (54.8)	
Adequate	70 (45.2)	
Locoregional treatment		
Inadequate	14 (9.0)	
Adequate	141 (91.0)	

G1, Well differentiated; G2, Moderately differentiated; G3, Poorly differentiated; N, Lymph node metastasis; T, Tumour size

**Table 2.** Univariate and multivariate Cox regression for DMFS of patients with HER2 tumours (*n* = 155).

	Univariate		Multivariate	
Factor	HR (95% CI)	*p*	HR (95% CI)	*p*
Trastuzumab exposition				
No	1			
Yes	0.362 (0.167–0.784)	0.010		
Trastuzumab exposition				
≤292 days/no	1		1	
>292 days	0.297 (0.127–0.698)	0.005	0.284 (0.121–0.668)	0.004
Trastuzumab exposition				
<6 months/no	1			
≥6 months	0.333 (0.147–0.756)	0.009		
Trastuzumab exposition				
<12 weeks/no	1			
≥12 weeks	0.349 (0.158–0.770)	0.009		
Trastuzumab exposition				
≤9 weeks/no	1			
>9 weeks	0.329 (0.149–0.726)	0.006		
St. Gallen Consensus treatment				
Inadequate	1			
Adequate	0.503 (0.222–1.137)	0.099		
pN				
N−	1		1	
N+	2.226 (1.061–4.671)	0.034	2.370 (1.128–4.981)	0.023

**Table 3.** Univariate and multivariate Cox regression for OS of patients with HER2 tumours (*n* = 155).

	Univariate		Multivariate	
Factor	HR (95% CI)	*p*	HR (95% CI)	*p*
Trastuzumab exposition				
No	1			
Yes	0.375 (0.160–0.882)	0.025		
Trastuzumab exposition				
≤292 days/no	1		1	
>292 days	0.202 (0.069–0.594)	0.004	0.203 (0.069–0.596)	0.004
Trastuzumab exposition				
<6 months/no	1			
≥6 months	0.249 (0.092–0.670)	0.006		
Trastuzumab exposition				
<12 weeks/no	1			
≥12 weeks	0.279 (0.110–0.705)	0.007		
Trastuzumab exposition				
≤9 weeks/no	1			
>9 weeks	0.264 (0.104–0.668)	0.005		
St. Gallen Consensus treatment				
Inadequate	1			
Adequate	0.278 (0.095–0.815)	0.020		
pN				
N0/N1	1		1	
N2/N3	2.555 (1.166–5.595)	0.019	2.530 (1.155–5.538)	0.020

**Table 4.** Univariate and multivariate Cox regression for DFS of patients with HER2 tumours (*n* = 155).

	Univariate		Multivariate	
Factor	HR (95% CI)	*p*	HR (95% CI)	*p*
Trastuzumab exposition				
No	1			
Yes	0.451 (0.228–0.891)	0.022		
Trastuzumab exposition				
≤292 days/no	1			
>292 days	0.372 (0.178–0.778)	0.009		
Trastuzumab exposition				
<6 months/no	1			
≥6 months	0.396 (0.193–0.811)	0.011		
Trastuzumab exposition				
<12 weeks/no	1			
≥12 weeks	0.398 (0.197–0.804)	0.010		
Trastuzumab exposition				
≤9 weeks/no	1		1	
>9 weeks	0.375 (0.186–0.757)	0.006	0.353 (0.175–0.714)	0.004
St. Gallen Consensus treatment				
Inadequate	1			
Adequate	0.448 (0.210–0.959)	0.039		
pN				
N−	1		1	
N+	2.018 (1.042–3.908)	0.037	2.183 (1.124–4.237)	0.021
